# Coordinated Regulation of ATF2 by miR-26b in γ-Irradiated Lung Cancer Cells

**DOI:** 10.1371/journal.pone.0023802

**Published:** 2011-08-25

**Authors:** Himanshu Arora, Rehana Qureshi, Ae-Kyung Park, Woong-Yang Park

**Affiliations:** Department of Biomedical Sciences, Biochemistry and Molecular Biology, Seoul National University College of Medicine, Seoul, Korea; Ajou University, Korea

## Abstract

MicroRNA regulates cellular responses to ionizing radiation (IR) through translational control of target genes. We analyzed time-series changes in microRNA expression following γ-irradiation in H1299 lung cancer cells using microarray analysis. Significantly changed IR-responsive microRNAs were selected based on analysis of variance analysis, and predicted target mRNAs were enriched in mitogen-activated protein kinase (MAPK) signaling. Concurrent analysis of time-series mRNA and microRNA profiles uncovered that expression of miR-26b was down regulated, and its target activating transcription factor 2 (ATF2) mRNA was up regulated in γ-irradiated H1299 cells. IR in miR-26b overexpressed H1299 cells could not induce expression of ATF2. When c-Jun N-terminal kinase activity was inhibited using SP600125, expression of miR-26b was induced following γ-irradiation in H1299 cells. From these results, we concluded that IR-induced up-regulation of ATF2 was coordinately enhanced by suppression of miR-26b in lung cancer cells, which may enhance the effect of IR in the MAPK signaling pathway.

## Introduction

MicroRNAs are transcribed by RNA polymerase II and bind to the 3′ untranslated region (UTR) to suppress translation of target mRNAs [1]. At the posttranscriptional level, microRNAs are involved in many biological processes, including development [2], proliferation, cell death [3], and tumorigenesis [4]. Many studies have analyzed the transcriptional regulation of mRNAs and microRNAs in γ-irradiated cells to understand cellular responses to ionizing radiation (IR) [5], [6], [7].

The mitogen-activated protein kinase (MAPK) pathway plays an important role in various biological processes, such as apoptosis, proliferation, differentiation, WNT signaling, and p53 signaling. MAPK signaling is often deregulated in human cancers, leading to uncontrolled cell proliferation and survival [8]. IR can induce activation of MAPK pathways to control cell survival in a cell type-dependent manner [9]. The IR responsive activation of MAPK signaling pathways is related to cell proliferation [10].

Most cellular signaling pathways can be regulated by transcriptional and posttranslational control of genes. The microRNAs miR-7, miR-4, miR-79, miR-2, and miR-11 are involved in Notch signaling pathways by targeting the regulatory sequence motifs in the 3′ UTR of target genes [11]. miR-15 and miR-16 are involved in the Nodal signaling pathway [12]. Nuclear factor of kappa light polypeptide gene enhancer in B-cells 1, a DNA damage-signaling mediator, is regulated by miR-9 and let-7 g in response to IR in lung cancer cell lines [7]. In the present study, we examined the time-series expression profile of microRNAs in γ-irradiated lung cancer cell lines. We tried to identify IR-responsive microRNAs that regulate expression of MAPK signaling genes through concurrent analysis of microRNA and mRNA profiles. We demonstrated the coordinated regulation of activating transcription factor 2 (ATF2), which is encoded by a MAPK signaling gene, by miR-26b in response to IR.

## Results

**Figure 1:**
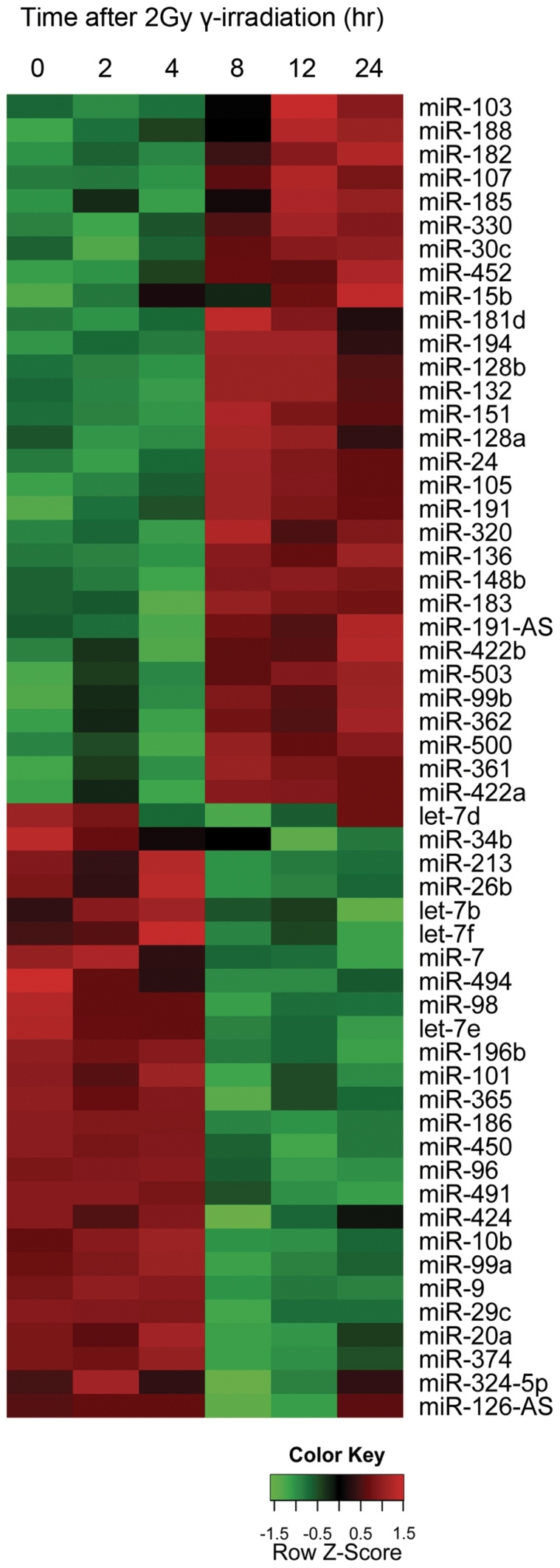
Heatmap illustrating expression of microRNAs in response to γ-irradiation in H1299 cells. Reverse transcribed small RNAs from each time point were labeled with Cy5. The color code represents the relative expression of indicated microRNAs for each time point. A list of all microRNAs is available in Table S1.

To understand posttranscriptional control of cellular responses to IR by microRNAs, the genome-wide expression profile of microRNA was examined in H1299 human lung cancer cells at 0, 4, 8, 12, and 24 hours after treatment with 2Gy of γ-radiation. The microRNA expression profile was analyzed by one-way analysis of variance (ANOVA) to select IR-responsive microRNAs. Among 328 human microRNAs on the microarray, the expression of 56 (17.1%: 30 up-regulated and 26 down-regulated) was significantly changed in H1299 cells (p<0.05; Figure 1 and Table S1). Prominent changes were observed at 8 hours after γ-irradiation in most of the IR-responsive microRNAs.

To explore the physiological meaning of IR-responsive microRNA, we listed predicted target mRNAs of IR-responsive microRNAs and the enriched signaling pathways were selected based on enrichment and statistical analysis of predicted target mRNA by DIANA-microT-3.0. Among the listed signaling pathways, we focused on the top 10 pathways based on the statistical significance (Table 1). We especially chose the MAPK signaling pathway for further analysis because this signaling pathway is essential for survival in response to DNA damage [13].

**Table 1:** Enrichment analysis for signaling pathways on target mRNAs of IR-responsive miRNAs.

KEGG pathways	-ln[*p* value]*
Ribosome	25.8
MAPK signaling pathway	23.5
Axon guidance	22.8
Focal adhesion	20.7
Oxidative phosphorylation	20.1
Ubiquitin mediated proteolysis	17.9
TGF-beta signaling pathway	17.7
Adherens junction	17.2
Wnt signaling pathway	16.9
Regulation of actin cytoskeleton	16.2

*p value based on DIANA analysis.

**Figure 2:**
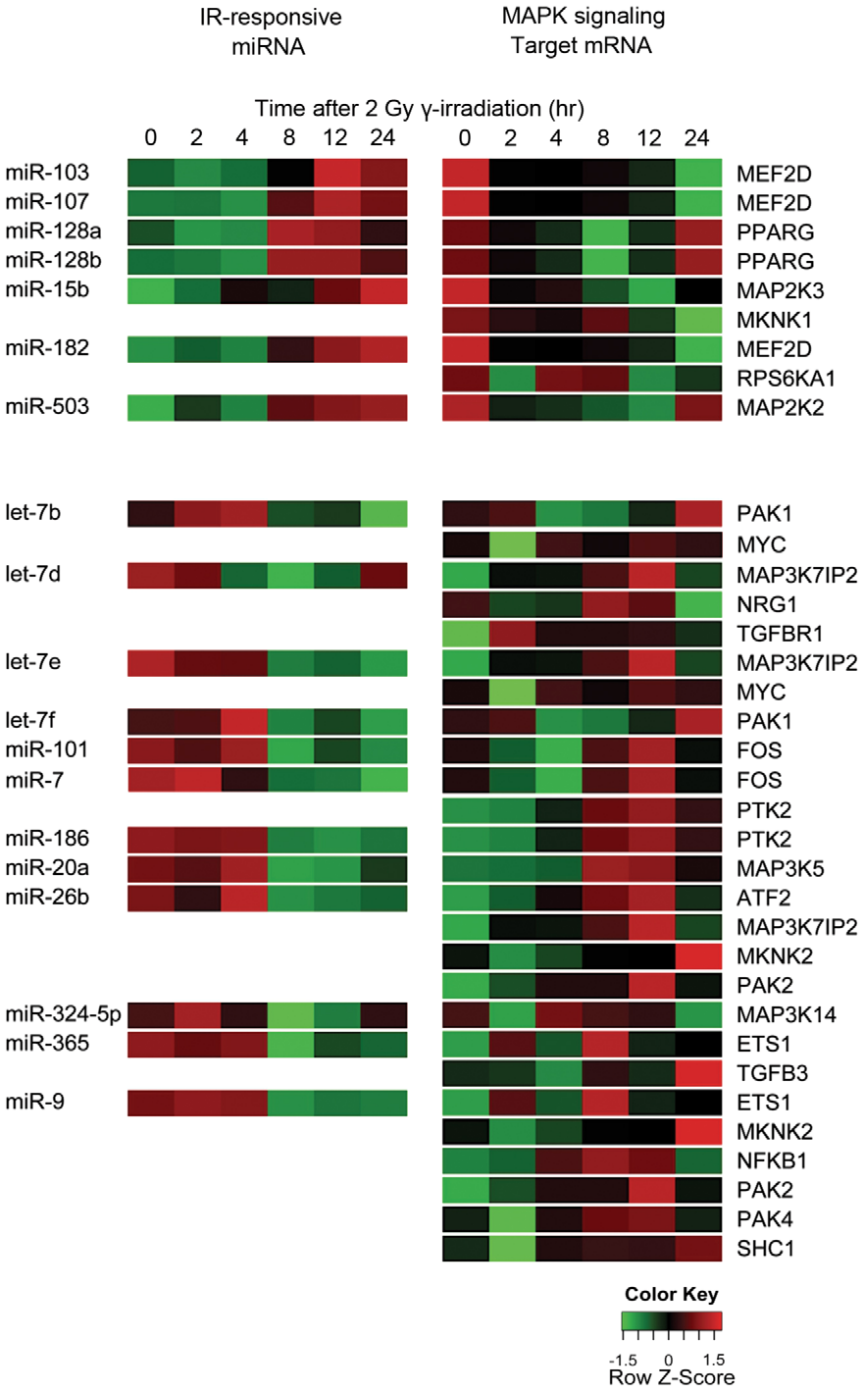
Heatmap illustrating the pairs of microRNAs and target mRNAs for the MAPK signaling pathway in response to γ-irradiation in H1299 cells.

To validate regulation of the MAPK signaling pathway by IR-responsive microRNAs, we meta-analyzed mRNA expression profiles of the same γ-irradiated H1299 cells from our published datasets [14]. In concurrent analysis of target mRNA and IR-responsive microRNA, we applied two criteria: 1) statistically significant changes (p<0.05) in mRNA expression upon γ-irradiation by ANOVA analysis and 2) the high inverse correlation value (r<−0.4) between mRNA and microRNA expression. As summarized in Figure 2 and Table S2, we identified 35 pairs of IR-responsive microRNAs and target mRNAs, including 19 microRNAs and 23 non-overlapping mRNAs for MAPK signaling pathway genes in H1299 cells.

**Figure 3:**
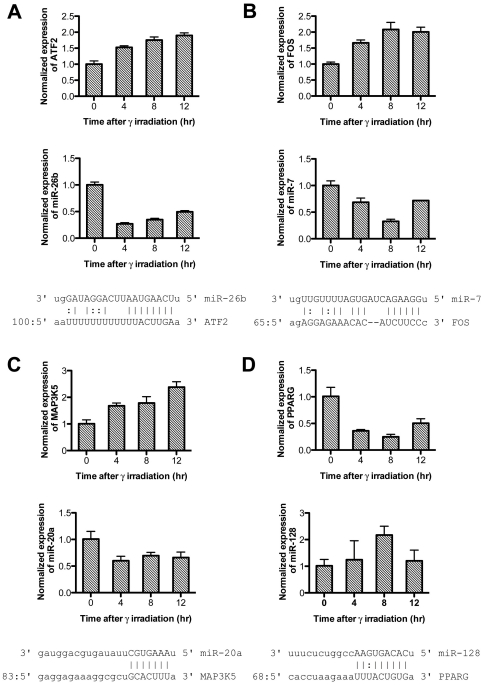
Expression patterns of IR-responsive microRNAs and MAPK signaling target mRNAs in γ-irradiated H1299 cells. The expression of four pairs of microRNA and target mRNA such as (A) miR-26b:ATF2, (B) miR-7:FOS, (C) miR-20a:MAP3K5, and (D) miR-128:PPARG) were quantitated using real-time reverse transcription-polymerase chain reaction (RT-PCR) at the indicated time. The values were normalized with glyceraldehyde 3-phosphate dehydrogenase (GAPDH) mRNA for target mRNAs and U6B small RNA for microRNAs. All values are presented as means ± standard deviation (SD) from triplicate experiments.

We validated the expression patterns of IR-responsive microRNAs and target mRNAs for the MAPK signaling pathway. Among 35 pairs, we selected and analyzed four (miR-26b: ATF2, miR-7: FOS, miR-20a: MAP3K5, and miR-128: PPARG) pairs by reverse transcription-polymerase chain reaction (RT-PCR; Figure 3A,B, C and D). As detected in microarray datasets (Figure 2), we found that ATF2, FOS, and MAP3K5 were up regulated and PPARG was down regulated upon IR exposure. MicroRNAs such as miR-26b, miR-7, and miR-20a were down regulated, and miR-128 was up regulated upon IR exposure. By real-time RT-PCR, we demonstrated that the expression patterns of selected IR-responsive microRNAs and target mRNAs were well matched with those of the microarray expression data.

**Figure 4:**
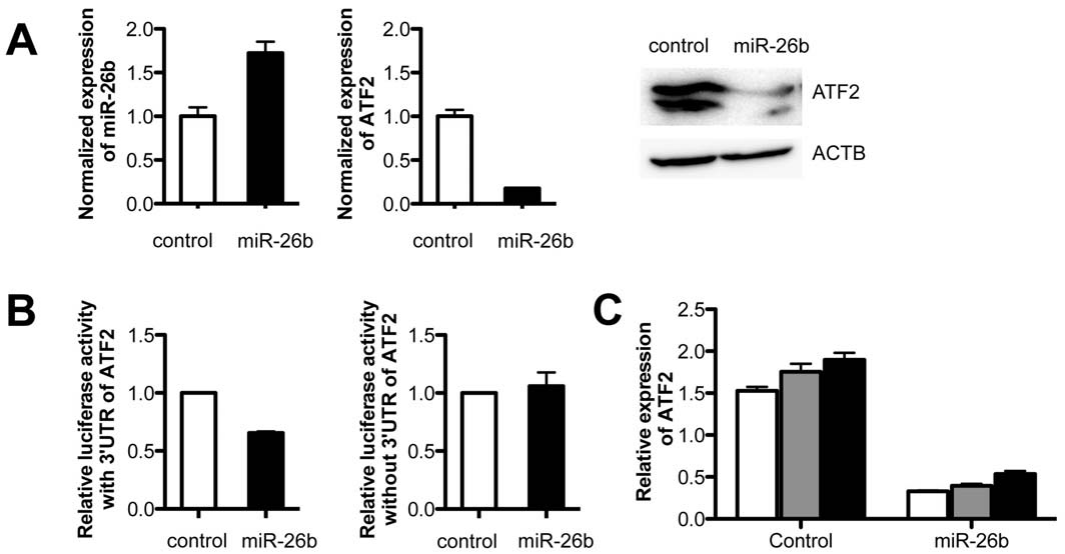
Suppression of activating transcription factor 2 (ATF2) by miR-26b. (A) In miR-26b transfected H1299 cells, the expression of microRNA was confirmed by real-time RT-PCR. The expression of ATF2 mRNA in miR-26b transfected cells was measured by real-time RT-PCR. The relative ATF2 expression levels were normalized against GAPDH and presented as mean ± SD from triplicate experiments. The protein level of ATF2 was also examined by western blot in microRNA-transfected cells. (B) Cells were transfected with the empty renilla luciferase reporter gene (psiCHECK2) or the reporter gene fused to the ATF2 3′ UTR. In addition, the cells were co-transfected with miR-26b or without miR-26b; Results are expressed as relative light units (RLU) and were normalized with the luciferase activity expressed constitutively by the psiCHECK2 vector. (C) The relative expression of ATF2 in miR-26b transfected and IR exposed cells at 4 (white), 8 (grey) and 12 (black) hours respectively.

Down-regulated IR-responsive microRNAs may augment the function of target mRNAs. To test the relationship between down-regulated IR-responsive microRNAs and target mRNAs, we selected the pair of ATF2 and miR-26b among 35 pairs to demonstrate coordinated regulation between microRNAs and target mRNAs upon IR exposure. One predicted target was identified for miR-26b at position 112–118 of the ATF2 3′ UTR, as shown in Figure 3A. Overexpression of miR-26b in H1299 cells could suppress the expression level of ATF2 mRNA. In addition, the protein level of ATF2 was decreased in miR-26b-overexpressed cells (Figure 4A). In luciferase assays, miR-26b suppressed the translation of luciferase in constructs with the 3′ UTR of ATF2, but not those without the 3′UTR (Figure 4B). The suppressive effect of miR-26b on ATF2 was also observed in γ-irradiated H1299 cells (Figure 4C), which was sustained until 12 hours after IR exposure.

**Figure 5:**
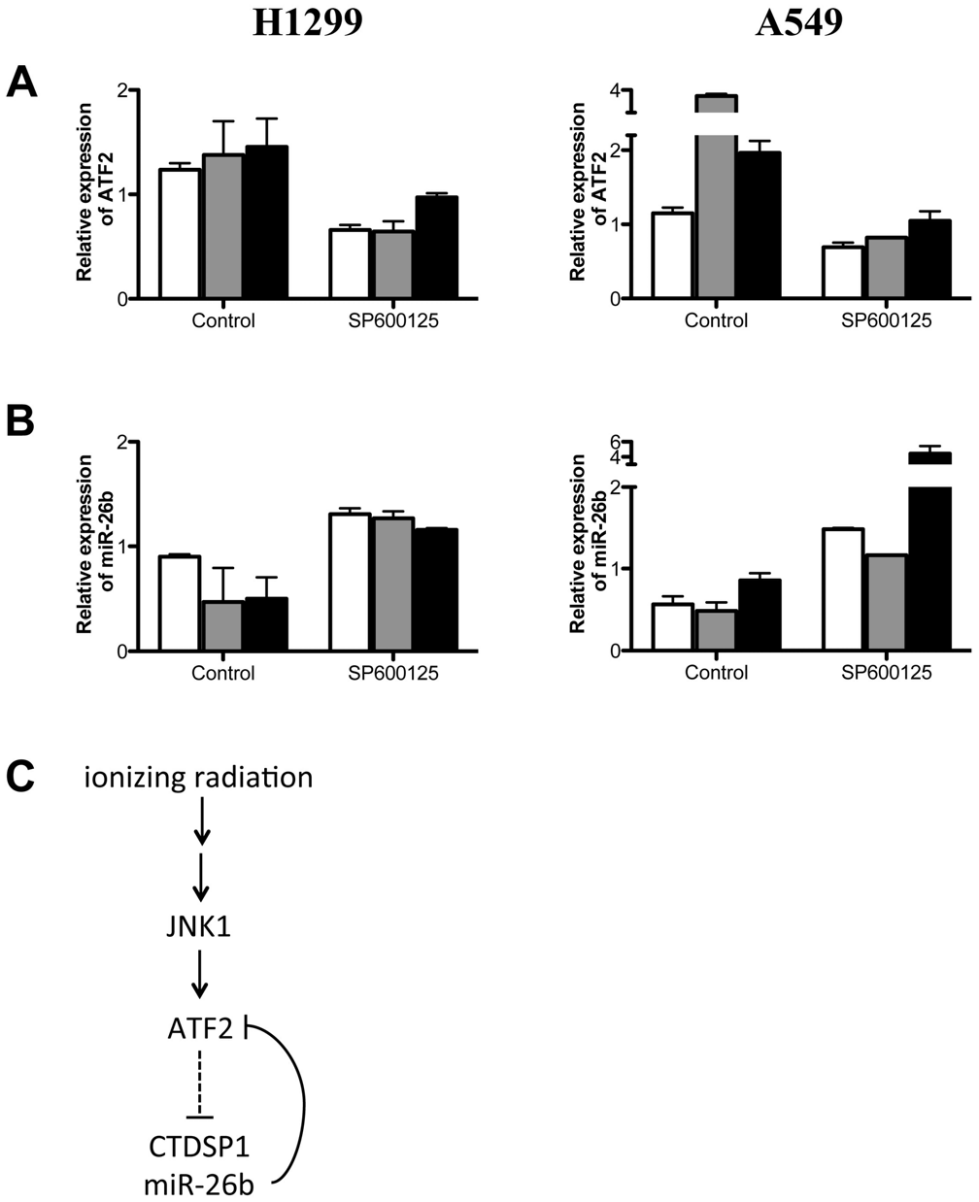
Effect JNK inhibitor on the expression of miR-26b in response to ionizing radiation (IR). H1299 and A549 cells were treated with 10 µM SP600125 for 30 minutes, and then exposed to IR. The relative expressions of ATF2 mRNA (A) and miR-26b (B) were normalized to the expression level of control at 0 hr in both of control and SP600125-treated cells at 4 (white), 8 (grey) and 12 (black) hours. (C) Ionizing radiation induced the expression of ATF2, which down-regulated the expression of miR-26b in γ-irradiated lung cancer cells.

Next, we wanted to confirm the effect of MAPK signaling on down-regulation of miR-26b in γ-irradiated cells. We inhibited the MAPK signaling pathway using SP600125, a c-Jun N-terminal kinase (JNK) inhibitor, in γ-irradiated H1299 cells. Treatment with SP600125 did not change the basal expression level of ATF2; however, induction of ATF2 upon γ-irradiation was markedly blocked in SP600125-treated H1299 cells until 12 hours after IR exposure (Figure 5A). Expression of ATF2 requires activation of MAPK signaling, which was inhibited at JNK by the chemical inhibitor. Conversely, expression of miR-26b was induced by treatment with SP600125 in H1299 lung cancer cells (Figure 5B). The effects of SP600125 on the expression of ATF2 mRNA and miR-26b were also confirmed in A549 lung cancer cell line (Figure 5).

## Discussion

Cellular responses to exogenous stimulation can be monitored by alterations in gene expression, including expression of microRNAs. IR can induce progressive changes in cell survival, growth, and proliferation by affecting gene expression. Previous reports have suggested that radiation can change the expression pattern of genes [15], [16]. We analyzed microRNA profiles to understand the mechanism of microRNA-mediated cellular responses to IR, and to identify regulation of the MAPK signaling pathway by IR-responsive microRNAs. In the present study, we have elucidated the JNK-mediated transcriptional suppression of miR-26b in γ-irradiated cells, for which microRNA can suppress the translation of target ATF2 mRNA, a member of the MAPK signaling pathway. From these findings, we suggest that the cellular response to IR is coordinately regulated by the interaction between the MAPK signaling pathway and microRNA.

ATF2 is a cAMP-response element-binding (CREB) protein with a basic leucine zipper (bZIP) domain, through which ATF2 interacts with other bZIP proteins such as JUN, FOS, CREB, and ATF1 [17], [18]. DNA damage and pro-inflammatory cytokines can induce activation of ATF2 transcriptional activity by JNK [19]. The role of diverse signaling in activation of ATF2 is also illustrated by heterodimeric partners of ATF2, which are also activated in a stimulus-specific manner. Thus, a particular stimulus can lead to different ATF2 complexes, thereby activating or repressing distinct sub-sets of target genes [20].

miR-26b is an intronic microRNA residing in intron IV of CTDSP1, C-terminal domain small phosphatase 1. The transcriptional control of host CTDSP1 mRNA is not fully understood, but many putative binding sites exist for transcription factors such as CREB in ENCODE Transcription Factor Binding Analysis [21]. ATF2 could suppress transcription of target genes through dimerization with other bZIP transcription factors. Overexpression of bZIP proteins such as ATF2 and CREB altered the gene expression in human myometrial cells [22]. Meta-analysis on this microarray datasets in GEO (GSE1059) revealed down-regulation of CTDSP1 in ATF2-overexpressed cells. We need further study regarding the transcriptional control of miR-26b by ATF2 in lung cancer cells; however, JNK activity and expression of ATF2 repressed expression of miR-26b is performed in the current study.

Deregulation of the MAPK signaling pathway can be induced by IR-induced DNA damage [23]. In the present study, it was found that MAPK signaling is induced in γ-irradiated H1299 cells, which might mediate the survival of H1299 lung cancer cells upon IR exposure. Furthermore, activation of MAPK signaling led to down-regulation of miR-26b, which supported the maintenance of ATF2 activity in turn. From these results, we could demonstrate that exposure of H1299 lung cancer cells to IR induced MAPK signaling followed by suppression of miR-26b expression, which led to the escape of ATF2 mRNA from posttranslational suppression by miR-26b. We propose that miR-26b mediates coordinate regulation of ATF2 and the MAPK signaling pathway in response to IR.

## Materials and Methods

### Cell culture

H1299 human lung cancer cells were maintained in RPMI 1640 and A549 cells were cultured in Dulbecco's Modified Eagle's medium (DMEM, Sigma Aldrich, St Louis, MO, USA) supplemented with 10% fetal bovine serum, 100 U/ml penicillin, 100 µg/ml streptomycin, and 2 mM L-glutamine [both cell lines were purchased from ATCC]. The cultured cells were either exposed to 2 Gy of radiation using a 4-MV linear accelerator (Clinac 4/100; Varian, Palo Alto, CA, USA) or left unirradiated as a negative control. The specific JNK inhibitor SP600125 was purchased from Santa Cruz Biotechnology (Santa Cruz, CA, USA). H1299 cells were incubated with 10 µM SP600125 for 30 min, and then exposed to IR (2 Gy) followed by total RNA isolation at indicated times.

### MicroRNA microarray

MicroRNA from each cell line was extracted using the mirVana microRNA isolation kit (Ambion, Austin, TX, USA) according to the manufacturer's protocols. Purified microRNAs were labeled using the mirVana microRNA Array Labeling Kit and coupled to the Cy5 Post-Labeling Reactive Dye (Amersham, GE Healthcare Bio-Sciences, Piscataway, NJ, USA). The labeled samples were washed and hybridized in duplicate to mirVana microRNA Bioarrays (Ambion) using the mirVana microRNA Bioarray Essentials Kit. Fluorescence intensities were processed and measured using the GeneChip scanner 3000 7G (Agilent Technologies, Santa Clara, CA, USA). The levels of microRNA hybridization were determined using GenePix Pro 6.0 software as recommended by the manufacturer. The background-adjusted intensity for each microRNA was subjected to a global variance stabilization normalization procedure [24]. All data is MIAME compliant and the raw data has been deposited in a MIAME compliant database (GEO)(accession number - GSE30075).

### Statistical and bioinformatics analysis

To identify microRNAs for which expression levels changed significantly throughout the time-course, we used one-way ANOVA analysis. Considering the correlation structure of within-array replicates [25] in mirVana microRNA Bioarrays, we performed one-way ANOVA analysis on 328 human microRNAs. DIANA (http://diana.cslab.ece.ntua.gr/), which integrates human and mouse microRNAs into pathways to predict microRNA targets [26], was performed initially to identify pathways.

### RNA preparation and quantitative real-time PCR

Total RNA was extracted from cell lines using the TRIzol method, and then reverse transcribed to complementary DNA using Superscript II reverse transcriptase (Invitrogen, Carlsbad, CA, USA) and oligo-(dT)12–18 primers according to the manufacturer's protocol. The quantitative RT-PCR for indicated genes was performed in a reaction mixture containing SYBR Premix Ex Taq (Takara Bio Inc., Shiga, Japan). Quantitation of microRNAs was performed using TaqMan microRNA assays (Applied Biosystems, Foster City, CA, USA) according to the manufacturer's protocol. Samples were analyzed using the ABI PRISM 7000 sequence detection system (Applied BioSystems). All PCRs were performed in triplicate, and the specificity of the reaction was determined by melting curve analysis at the dissociation stage. We synthesized specific primers for ATF2 (forward: 5′-AGATTTATTAATTTTTCTGTGCTCAA-3′; reverse: 5′ ACACCCCCATTTATTAAAACACC-3′), FOS1 (forward: 5′-TGTGTTCCTGGCAATAGTGTG-3′; reverse: 5′-CAATGAACATTGATGTTGAAGAAA-3′), MAP3K5 (forward: 5′-GCAGCAGCTATTGCACTTCA-3′; reverse: 5′-TGGTCACATTTTGGTTTTGTTC-3′) and PPARG (forward: 5′-CCTGCAGGAGATCTACAAGGA-3′; reverse: 5′-GGTGTCAGATTTTCCCTCAGA-3′). The relative quantitative method was used for the quantitative analysis. The calibrator was the averaged ΔCt from the untreated cells. The endogenous control was glyceraldehyde 3-phosphate dehydrogenase (GAPDH) for genes and U6B for microRNAs.

### Western blotting

Cells were harvested and lysed in NP-40 buffer containing phenylmethylsulfonyl fluoride and Protease Inhibitor Cocktail (Sigma, St. Louis, MO, USA). Protein extracts were then separated by sodium dodecyl sulfate-polyacrylamide gel electrophoresis, and then transferred to polyvinylidene fluoride membranes (Bio-Rad, Hercules, CA, USA). Membranes were incubated with an ATF2 antibody (1∶1000; Santa Cruz Biotechnology Inc.) in Tris-buffered saline Tween 20 buffer with non-fat dry milk, and then incubated with horseradish peroxidase-conjugated secondary antibody (dilution 1∶5000; Bio-Rad). Immunoreactive bands were visualized using the West-Q-Chemiluminescent Substrate Kit Plus (BIOTANG, Waltham, MA, USA).

### Constructs, transfection, and luciferase assay

The precursor of miR-26b was cloned into pcDNA3 (Invitrogen) by genomic DNA PCR with primers (forward: 5′-CCGGAATTCCGGATGGGAATTGGATACAT-3′; reverse: 5′-ATTGCGGCCGCAGCTACCCTGACCACTGCTGC-3′). The 3′ UTRs of ATF2 were cloned downstream of the Renilla luciferase gene in the psiCHECK2vector (Promega, Fitchburg, WI, USA). The construct was transfected using FuGENE HD reagent (Roche, Basel, Switzerland) for real-time RT-PCR, Western blotting, and luciferase assays. Luciferase assays were performed using the Dual-Luciferase assay kit (Promega). Normalization of Renilla expression was performed using firefly luciferase present in the psiCHECK2 vector.

## Supporting Information

Table S1 **List of selected microRNAs from H1299 cells.**
(XLSX)

Table S2 **Selected mRNA:microRNA pairs of the MAPK signaling pathway based on the enrichment analysis.**
(XLSX)
